# First encounters of the microbial kind: perinatal factors direct infant gut microbiome establishment

**DOI:** 10.20517/mrr.2021.09

**Published:** 2022-03-01

**Authors:** Kevin Linehan, Eugene M. Dempsey, C. Anthony Ryan, R. Paul Ross, Catherine Stanton

**Affiliations:** ^1^Teagasc Food Research Centre, Moorepark, Fermoy, Co. Cork P61 C996, Ireland.; ^2^APC Microbiome Ireland, Biosciences Institute, University College Cork, Lee Maltings, Cork, Cork T12 YT20, Ireland.; ^3^School of Microbiology, University College Cork, Cork T12 YN60, Ireland.; ^4^Department of Paediatrics & Child Health and INFANT Centre, University College Cork, Cork T12 YN60, Ireland.

**Keywords:** Gut microbiome, neonatal microbiota, diseases, antibiotics, early programming, mode of birth, perinatal factors

## Abstract

The human gut microbiome harbors a diverse range of microbes that play a fundamental role in the health and well-being of their host. The early-life microbiome has a major influence on human development and long-term health. Perinatal factors such as maternal nutrition, antibiotic use, gestational age and mode of delivery influence the initial colonization, development, and function of the neonatal gut microbiome. The perturbed early-life gut microbiome predisposes infants to diseases in early and later life. Understanding how perinatal factors guide and shape the composition of the early-life microbiome is essential to improving infant health. The following review provides a synopsis of perinatal factors with the most decisive influences on initial microbial colonization of the infant gut.

## INTRODUCTION

The microbiome depicts the microbiota and their “theatre of activity”, encompassing the combined nucleic acids (including viruses and bacteriophages), structural components and microbial metabolites related to the microbiota^[1]^. The gut microbiota describes the trillions of microbial cells (bacteria, archaea, protists, and fungi) occupying the intestine, with Bacteroidetes and Firmicutes accounting for 90% of the total bacterial inhabitants and Actinobacteria, Cyanobacteria, Fusobacteria, Proteobacteria and Verrucomicrobia constituting the remaining 10%^[2]^. These gut microbiota engage in a plethora of host biological responses, including the regulation of immunity^[3]^, energy homeostasis, protection against pathogens and bioactive metabolite production^[4]^. Furthermore, the disparities in the intestinal microbiota have been linked to disease states such as metabolic disorder^[5]^, cancer^[6]^, and cardiovascular disease^[7]^. The initial formation of the gut microbiota is essential in maintaining intestinal homeostasis and symbiosis in neonates, shaping and guiding the development of the immune and nervous systems during a critical developmental window^[8]^. Although the mechanism at present is not fully elucidated, the current consensus is that a disrupted microbiota in early life can leave a lasting footprint on health in early life and beyond^[9]^. The first encounters of the neonatal gut with microbes are directed by intrinsic and extrinsic factors, collectively named “perinatal factors”. These consist of maternal nutrition, antibiotic use, gestational age, genetics and mode of delivery^[10]^. Early-life gut microbiome imbalances predispose infants to colonisation by opportunistic pathogens, such as *Enterococcus*, *Enterobacter*, *Clostridium*, and *Klebsiella *species. Furthermore, infant gut dysbiosis can lead to impaired growth and an increased risk of sepsis and necrotizing enterocolitis (NEC), especially in preterm new-borns^[11]^. Longer-term consequences of perturbed microbial colonization include allergies, asthma, metabolic syndrome, diabetes and inflammatory bowel disease^[12]^. Therefore, it is essential to elucidate the extent to which perinatal factors may influence the microbial bond between mother and infant. Further understanding of how this intricate relationship may be influenced by perinatal factors is imperative to optimise mother and infant health in the perinatal period and inform the adequate type and timing of therapeutic interventions. In this short review, we discuss the core microbes which initially colonize the infant gut and decipher the influence of perinatal factors on directing these first encounters.

## CORE INFANT GUT MICROBIOME

The first encounters of microbes with the infant intestine represent the *de novo* assembly of a complex microbial community^[13]^. In contrast to adults, the gut microbiome in early life is unstable, highly dynamic and low in diversity^[14]^. At birth, the infant gut is an aerobic environment, inhabited by *Escherichia *and *Enterococcus*. These facultative bacteria thrive on residual oxygen present, resulting in a lowered redox potential and thus shifting the infant gut to an anaerobic environment. Obligate anaerobes, including Firmicutes such as *Clostridia*, Bacteroidetes and the bedrock of the infant gut microbiome, bifidobacteria, begin to flourish at this point^[15]^. Bifidobacteria constitute up to 37% of the infant gut microbiota in early life^[16]^. Along with *Veillonella*, *Streptococcus*, *Citrobacter*, *Escherichia*, as well as *Bacteroides *and *Clostridium*, they dominate the core infant gut microbiome^[17]^. In Table 1, we summarize the characteristics and functions of these pioneering microbes in the infant gut. Despite large variance at the intra-individual level, a recent multi-population cohort meta-analysis revealed the existence of specific taxonomic patterns or infant community state types (ICSTs)^[29]^. This study subdivided ICSTs into four macro groups, < 1 month of age, between 1 and 6 months of age, between 6 and 12 months of age and between 1 and 3 years of age. Higher microbial complexity in samples from infants aged between 12 and 36 months compared to those from children less than one-month-old was observed. This increase in biodiversity appeared to have caused a reduction in the relative abundance of the *Bifidobacterium *genus in favour of *Bacteroides*, *Feacalibacterium*, *Blautia *and *Ruminococcus *genera. ICSTs are further modulated by birth mode and feeding type, which will be discussed later in this review.

**Table 1:** Characteristics, functions and metabolites produced by the core infant gut microbiome members

**Microbes**	**Key functions & characteristics in the infant gut**	**Microbial metabolites produced**	**Refs.**
Bifidobacteria	Saccharolytic degradation of diet-derived glycans and host-provided carbohydrates, known as host glycans, including mucins and human milk oligosaccharides (HMOs)	γ-aminobutyric acid (GABA), conjugated linoleic acid (CLA), short chain fatty acids (SCFA), Group B vitamins (B1, B3, B6, B9, B12)	[18-20]
Genus *Bacteroides*	Metabolize HMOs, mucins and complex plant polysaccharides (starch, cellulose, xylans, and pectins). Proteolytic activity via extracellular proteases. Deconjugation of bile acids	GABA, vitamin K2, SCFA, Group B vitamins (B1, B2, B5, B6, B7, B9, B12)	[19-23]
Genus *Veillonella*	Produce propionate from end products of carbohydrate fermentation (e.g., lactate). Propionate displays anti-inflammatory features, influences glucose and energy homeostasis and increases insulin sensitivity	SCFA	[24]
Genus *Streptococcus*	Specific members of the genus *Streptococcus* form part of the core infant gut microbiota and are among the first established bacteria in the infant gut within the first 24 h following birth	Serotonin, GABA, histamine	[19]
Genus *Collinsella*	Present in large amounts when infant gut microbiota is dominated by bifidobacteria. Currently comprises six species isolated from human faeces and vaginal tract	Group B vitamins (B6)	[20,25,26]
Genus *Lactobacillus*	Vertical transmission of *Lactobacillus* species presents the origin of infant *Lactobacillus* microbiota component. It may be related to the HMO metabolism of infants	Serotonin, GABA, acetylcholine, histamine, CLA, SCFA, Group B vitamins (B1, B2, B7, B9, B12)	[19,20,27]
Genus *Akkermansia*	*A. muciniphila* is the sole intestinal representative in the human gut from early life. May ferment HMOs. The target for therapeutics aimed at increasing barrier function	Serotonin, SCFA	[28]

## PERINATAL FACTORS AND THE FIRST MICROBIAL ENCOUNTERS

**Figure 1:**
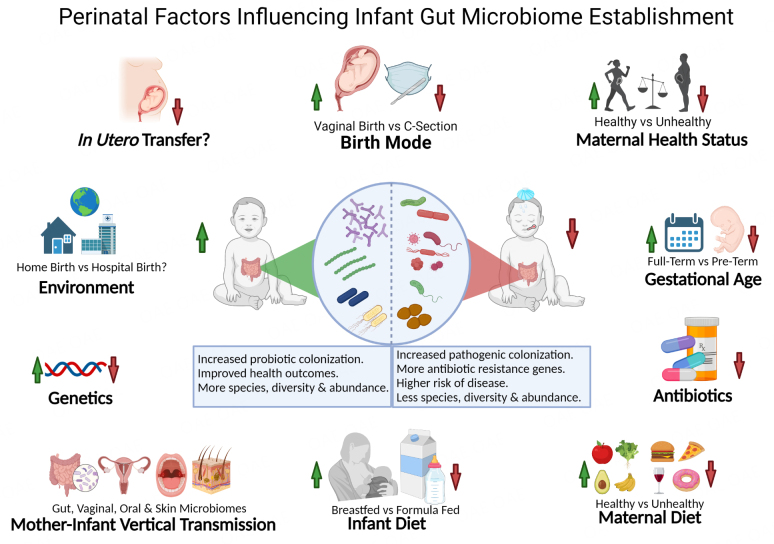
Perinatal factors influencing infant gut microbiome establishment. Graphic outlining the factors that contribute to early the initial infant gut microbiome. Created with BioRender.com.

In this section, we discuss the effect of perinatal factors on the initial colonization of the infant gut with microbes (see Figure 1). The majority of studies on this topic relate the relative abundance of microbes in fecal samples to the composition of the gut microbiota, thus any changes or differences in microbial content discussed herein are based upon changes/differences in the relative abundance of microbes unless otherwise stated. While this review will focus on the establishment of bacteria in the infant gut, we acknowledge that these factors may also influence the infant gut virome^[30]^, mycobiome and sporobiota^[31]^.

### Maternal health status during pregnancy

Maternal health during pregnancy is a key input in fetal health and infant development. Numerous studies have described aberrant gut colonization in infants born to mothers who suffered from elevated stress and cortisol levels throughout gestation^[32]^. Specifically, increased abundances of pathogenic bacteria Proteobacteria (*Escherichia*, *Enterobacter*, *Serratia*, *Citrobacter*, *Campylobacter*) and Firmicutes (*Veillonella *and *Finegoldia*) in addition to decreased abundances of beneficial bacteria such as lactic acid bacteria (*Lactobacillus*, *Lactococcus*, *Aerococcus*), Actinobacteria (bifidobacteria, *Collinsella*, *Eggerthella*) and *Akkermansia* have been noted^[32]^. While weight gain during pregnancy is a natural physiological response, excessive gestational weight gain (defined as 16 kg or above for women with BMI 19.8-25 or above 11.5 kg for women with BMI > 25^[33]^ has been reported to lead to the increased relative abundance of *Escherichia *and *Dorea *in vaginally born infants^[34]^. Metagenomic analysis has also shown that gestational weight gain impacts the function of the initial infant gut microbiome^[35]^. Excessive maternal weight gain during pregnancy has been associated with enrichment of phenylalanine, cysteine/serine, folate, thiamine, biotin, and pyridoxine synthesis pathways and bacterial glucose pathways in infants^[35]^. These effects are seen up to eight months post-partum, highlighting a longer-term impact on microbiota function. Affecting one in every seven live births worldwide^[36]^, gestational diabetes mellitus (GDM) presents the most frequent metabolic disorder that women encounter throughout gestation. The establishment of key pioneering taxa, important for establishing neonatal development, such as *Lactobacillus*, *Flavonifractor*, *Erysipelotrichaceae *and *Bacteroides *have shown to be decreased in infants born to mothers with GDM^[37]^. Interestingly, the level of dysbiosis is postulated to occur differentially depending on the severity of the GDM condition^[38]^. Both extremes of the reproductive age (typically < 17 years and > 35 years) are considered as the highest risks for infant mortality and detrimental health outcomes for mother and infant. Fetal and maternal complications of GDM include fetal growth restriction, preterm birth, chromosomal and congenital abnormalities, pre-eclampsia and C-section (CS) birth^[39-41]^.

### Maternal diet

Mounting evidence demonstrates the influence of a healthy maternal diet throughout the gestational period to optimize the acquisition of rich and diverse gut microbiota in neonates and improve long-term health outcomes^[42]^. While the quintessential prenatal diet is not known, it is recommended that pregnant women consume “a balanced diet with the appropriate distribution of the basic food pyramid groups”^[43]^. Regarding studies of the influence of maternal diet on infant gut microbiome establishment, it must be noted that notable heterogeneity pervades multiple areas of the studies presented to date. For instance, most studies utilize cohorts of overweight or obese women, which are not representative of a normal population. At present, there is no clear consensus on the periods of assessment and time points during pregnancy at which assessments are carried out. A systematic review by Maher *et al.*^[42]^ recently reported that inspection of detailed dietary data in pregnancy and its impact on the microbiome must be carried out in detail in a cohort that is indicative of a normal obstetric population. Bereft of this information, current findings from subgroups are challenging to decipher and highly variable. Finally, the authors noted that the methods of reporting dietary intake are subject to bias, suggesting that combining both Food Frequency Questionnaires and food diaries may be a method to circumvent this problem. With these limitations in mind, high-fat maternal diet has been repeatedly associated with detrimental effects on the infant gut microbiome^[42]^. High-fat maternal diet has been reported to be associated with the enrichment of *Enterococcus* and depletion in *Bacteroides*^[44]^. A recent study reported that diet in a healthy cohort of pregnant women was correlated to both maternal and neonatal microbiota at the time of birth, in a delivery mode-dependent manner^[45]^. Furthermore, the study indicated that fat intake in the form of saturated fatty acids and mono- and poly-unsaturated fatty acids (MUFA and PUFA) showed significant enrichment in Firmicutes phylum genera and depletion in Proteobacteria phylum genera in the offspring’s microbiota. A high-fat diet has been related to a higher abundance of Firmicutes and a concurrent increase in BMI^[46,47]^. Maternal intestinal permeability is higher throughout pregnancy^[48]^ and excess dietary intake is known to enhance intestinal permeability^[49]^, which may have implications on the vertical transmission of specific genera influencing early neonatal colonization. Prenatal diet has also been shown to modify the breast milk microbiome. High dietary intake of plant protein, fiber, and carbohydrates was associated with elevated *Staphylococcus*, *Bifidobacterium*, and *Lactobacillus* abundance^[50]^. The intake of food with high animal protein content and fats (n-3 PUFAs and MUFAs), exhibited a negative association with the abundance of *Enterococcus* and *Bifidobacterium* and a positive correlation with *Gemella*^[50]^ in the breast milk microbiome. Maternal diet may also modulate the human milk oligosaccharide (HMO) composition of breast milk^[51]^. Modulation of the HMO profile of breast milk by dietary manipulation may represent a method to manipulate the establishment of microbial species in the infant gut^[51]^. Further studies are required to elucidate this relationship fully.

### *In utero* microbiome transfer

Microbiome studies of the *in utero *environment highlight numerous limitations of current next-generation sequencing (NGS) methodologies. Contamination^[52]^ or the “kitome”^[53]^ represents a major issue when NGS and PCR-based approaches are applied to low-biomass samples, such as those encountered in the *in utero* environment^[54]^. Without stringent controls and method standardisation, these methodologies will amplify any DNA present in the sampled environment, regardless of bacterial viability^[55]^, thus, inaccurately reflecting the microbiota present in the sample^[56]^. Studies to date which have applied NGS methods informed by the potential for false-positive results have failed to detect a placental microbiome^[53,57-60]^. However, a well-controlled study by Edmond *et al.*^[61]^ indicated that the placenta represents a potential site of perinatal acquisition of *Streptococcus agalactiae *[group B *Streptococcus *(GBS)], a major cause of neonatal sepsis. Robust studies of amniotic fluid have failed to find a resident microbiome^[62-64]^. Indeed, *Enterobacter *and *Escherichia*, commonly identified contaminants in laboratory and extraction kit reagents^[65,66]^ have been reported as the most abundant genera detected in amniotic fluid^[67,68]^. Similarly, controlled studies have reported that the microbial content of meconium does not differ from negative controls^[69,70]^. A recent study claimed to have detected bacterial DNA and viable bacteria in the fetal intestine using 16S rRNA gene sequencing, qPCR, electron microscopy, and bacterial culture of *Micrococcus luteus*-related bacteria, which appeared to show adaptations to the fetal environment, re-igniting the controversy surrounding the existence of *in utero *microbiomes^[71,72]^. The studies presented thus far support the consensus that microbial colonization of the infant occurs at birth and the establishment of replicating microbial cells does not commonly occur in healthy pregnancies, devoid of clinical infections^[73]^. This is in agreement with Walter and Horneff^[74]^, who highlighted that there is no overlap between the bacterial taxa detected *in utero *in the sequencing studies of the *in utero *environment, with evident conformity between the bacterial taxa identified *in utero *and the controls.

### Birth mode

Throughout vaginal birth, the infant is primarily exposed to the maternal intestinal and vaginal microbiota^[75]^. Microbes of the birth canal such as *Lactobacillus*, *Prevotella* and *Sneathia* are typically detected in the infant gut of vaginally born infants. *Bacteroides*, *Escherichia*, *Bifidobacterium*, and *Parabacteroides* are also usually found^[76]^. These genera dominate the infant microbiota and comprise up to 68% of all microbes present four days after birth^[77]^. CS removes the infant’s exposure to maternal intestinal and vaginal microbes, thus blocking vertical transmission and is the perinatal factor with the most uniform effects on the infant gut microbiota across individuals and studies^[78]^. A ubiquitous feature of the CS-born infant’s gut microbiota is the low relative abundance of bifidobacteria and almost total lack of *Bacteroides*^[79]^. The relative abundance of *Enterococcus*, *Staphylococcus*, *Streptococcus*, *Klebsiella*, *Enterobacter*, *Propionibacterium*, and *Clostridium *is also higher in the CS born infant gut^[80]^. Many of these taxa are common representatives of endemic opportunistic pathogens responsible for nosocomial infections^[81]^ and are common to the maternal skin and hospital environment^[82]^. The type of CS undertaken may further modulate its effects on the infant gut microbiome. For instance, the skin and vagina are thought to be the source of the gut microbiota in emergency CS, whereas the skin is considered to be the predominant microbial origin of the gut microbiota in infants born by elective CS^[83]^. Recent studies have attempted to utilise orally delivered fecal microbiota transplantation (FMT) and oral administration of maternal vaginal microbiota to restore disturbed intestinal microbial colonisation in CS born infants^[84,85]^. Despite its recent inception, FMT has yielded encouraging results^[84]^, while vaginal transplantation has delivered less comprehensive findings^[85]^.

### Mother-infant vertical transmission

Following birth, infants appear to share 50% of microbial species in their gut with those found in the maternal gut, oral, vaginal, or skin microbiota indicating vertical transmission of microbes from mother to infant^[86]^. A study by Ferretti *et al*.^[86]^ reported that a significant proportion (50.7%) of the microbes present in the infant gut immediately post-partum, were transferred from the mother’s gut, vagina, oral cavity, or skin. The presence of these microbes was largely stable up to four months of age^[86]^. The mother’s gut accounted for the most significant proportion of microbes (22.1%), followed by the vagina (16.3%), the oral cavity (7.2%), and the skin (5%)^[86]^. The maternal gut microbiota represents the greatest maternal source of infant-acquired strains immediately post-partum, with most strains from Actinobacteria and Bacteroidetes^[16]^. Furthermore, a recent large scale meta-analysis of whole metagenomic shotgun sequencing data of maternal and infant fecal samples identified a set of 26 mother-infant shared species (species of *B. uniformis*, *B. vulgatus*, *B. longum*, *P. distasonis*, *P. merdae* confirmed at strain level) with high prevalence and relative abundance across cohorts studied^[87]^. The vaginal microbiome during gestation is dominated by *Lactobacillus *species, displaying decreased diversity and increased stability compared to non-pregnant women^[88]^. The vaginally delivered infant gut microbiome is dominated by *Lactobacillus*, *Prevotella*, *Atopobium* or *Sneathia* species, which are typical of the mother’s vaginal microbiome^[89]^ The vaginal microbiomes impact on the infant before birth remains largely unknown, however gestational changes that happen during pregnancy could be part of an adaptive response to promote the correct development and health of the fetus^[90]^. While the vaginal microbiome does contribute bacteria to the infant gut microbiome it may be that this contribution is minimal, in terms of overall abundance due to the low diversity of the vaginal microbiome during pregnancy^[81]^. The mother’s oral cavity and skin represent the other possible maternal sources of microbes. The contributions of microbes from these areas to the initial infant gut microbiome are significantly less than the mothers gut, vaginal and breast milk microbiomes. Furthermore, bacteria transferred from these sites colonize the infant gut in a transient manner^[91,92]^. However, significant microbial transfer from the mother’s skin and oral cavity to infants in later life is feasible, although additional studies are needed.

### Gestational age

Gestational age is one of the most significant influencers of gut microbiota formation^[93]^, defined as full-term (37-42 weeks), preterm (< 37 weeks) or post-term (> 42 weeks). The intestinal microbiota of the preterm infant is distinguished by delayed colonization, diminished species diversity and abundance. There is also a predilection to being colonized by pathogenic facultative anaerobes (e.g., *Enterobacter*, *Escherichia*, and *Klebsiella*) and decreased levels of commensal strictly anaerobic organisms (e.g., *Bifidobacterium*, *Bacteroides*, and *Clostridium*)^[94]^. Numerous studies have indicated a shared patterned progression of colonization by bacilli, Gammaproteobacteria and Clostridia in preterm infants^[95]^. In particular, *Enterococcus faecalis*, *Enterobacter cloacae*, *Staphylococcus epidermidis*, *E. coli*, *Klebsiella pneumoniae*, and *Klebsiella oxytoca *species are frequently found in the gut microbiota of preterm infants^[96]^. Preterm infants also encounter numerous distinctive environmental and host conditions, which can have deleterious effects on the establishment and subsequent formation of their gut microbiome. For instance, even before birth, between 25%-30% of preterm infants are exposed to microbes in the context of preterm premature rupture of membranes and intra-amniotic infection^[97]^. CS birth is also common in preterm birth cases; thus, colonization by the skin and environmental conditions rather than vaginal and rectal microbiota commonly occur^[78]^. Furthermore, exposure to the Neonatal Intensive Care Unit (NICU) has been related with a NICU-associated core microbiota composed of bacterial families *Enterobacteriaceae* (genera *Klebsiella* and *Escherichia *in particular) and Enterococcaceae^[98]^. A common NICU practice, respiratory support, has recently been shown to drive differences in microbiota development between extremely and very preterm infants^[98]^. Specifically, preterm infants exhibited higher colonization by facultative anaerobes and delayed colonization by obligate anaerobes^[98]^. As a result, with the shift in the ratio of facultative to obligate anaerobic bacteria, defense against pathogenic bacteria can often be impaired^[93]^. Finally, antibiotic administration in NICUs is commonplace for treating and preventing infections and sepsis^[93]^ and will be discussed in detail below.

### Antibiotics

In light of the ever-evolving antimicrobial resistance (AMR) crisis^[99]^, deciphering the implications of perinatal antibiotic usage on the establishment of the infant gut microbiome is paramount. Oral antibiotic use in pregnancy or intravenous antibiotic prophylaxis during delivery is common practice - especially for maternal GBS positivity, preterm premature rupture of the membranes, and/or as prophylaxis against wound infection in CS^[100]^. Vertical transmission of antibiotics and antibiotic-resistant microorganisms from mother to infant has been proposed to negatively impact the development and succession of the infant gut microbiota^[101]^. Antibiotic usage presents two potential problems in the perinatal period, through the transfer of antibiotics and/or AMR strains from mother to infant via *in utero *transfer or breastfeeding. The majority of broad-spectrum antibiotics (e.g., amoxicillin, gentamicin, vancomycin) used in the perinatal period can be readily transmitted to the fetus *in utero* via the placenta and umbilical vein due to simple diffusion and blood flow^[102]^. Because of the restricted activity of fetal hepatic drug-metabolizing enzymes compared with adults, the non-metabolized drug thus accumulates in the fetal tissues^[103]^. There is strong evidence that maternal antibiotic treatment throughout gestation reaches the infant in adequate amounts to potentially impact their resistome profiles^[104]^. Numerous studies have explored the effect of antibiotic use in pregnancy on infants’ intestinal microbiome composition and/or diversity. Reduced relative abundances of Actinobacteria, specifically *Bifidobacterium*^[104]^ and *Bacteroides*^[105] ^are commonly observed in infants whose mothers were treated with antibiotics prenatally or during delivery compared to those without. Increased relative abundances of Firmicutes and Proteobacteria are also consistently reported^[106]^. To date, most studies concentrate on the detrimental effects of antibiotics on the mother or fetus but lack exploration of the AMR aspects. There is also a pressing need for characterization of the species and strains harbouring these resistance genes^[107]^.

### Infant diet

Human breast milk is the “gold standard” nutrition for infant health and development and is considered the second integral source of microbes to the infant after the birth canal^[108]^. Harbouring > 700 bacterial species, breast milk ensures the consumption of up to ~800,000 bacteria daily^[109]^. HMOs modulate the immune system, prevent adhesion of pathogenic bacteria and act as metabolic substrates for gut bifidobacteria. In turn, bifidobacteria utilize their arsenal of membrane transporters and saccharolytic enzymes to cleave HMOs into their constituent monomers and internalize these or intact HMOs into the central catabolic pathways, leading to the main end products, lactate, acetate, formate, and 1, 2-propandiol^[110]^. This process aids in suppressing the growth of opportunistic pathogenic species within *Clostridiaceae*, *Enterobacteriaceae*, and *Staphylococcaceae*^[111]^. Multiple studies have isolated and identified bacterial strains, mostly *Bifidobacterium*, *Lactobacillus*, and *Staphylococcus*, which are common to both breast milk and infant faeces^[112]^. However, breastfeeding is not chosen/not possible in many circumstances. Exclusively formula-fed infants harbor a more diverse microbiota with lower abundances of HMO-utilizing *Bifidobacterium* species, often with increased abundances of *Clostridium* species (*C. difficile* and *C. perfringens*) and *Enterobacteriaceae* species (e.g., *E. coli*)^[113]^. While the gap between the composition of commercial formulas and breast milk is narrowing^[114]^, the gut microbiota of formula and breast-fed infants remain distinct^[35]^.

### Probiotics

Probiotics are defined by the FAO/WHO as “live microorganisms which when administered in adequate amounts confer a health benefit on the host”^[115]^. Probiotic supplementation in infants to ameliorate aberrant colonization, typically with *Bifidobacterium* and *Lactobacillus *strains, has yielded mixed results^[10]^. Probiotic usage for the prevention of NEC in preterm infants has yielded encouraging findings^[116]^. A recent meta-analysis concluded that *L. acidophilus *LB was the best supplementation option for reducing NEC risk in preterm infants^[117]^ A synbiotic is defined as “a mixture comprising live microorganisms and substrate(s) selectively utilized by host microorganisms that confers a health benefit on the host”^[118]^. A synbiotic consisting of short-chain galacto-oligosaccharides and long-chain fructo-oligosaccharides and the bifidobacterial strain *B. breve *M-16V, with a similar dosage to bacterial numbers of human milk, was reported to substantially increase levels of bifidobacteria in exclusively formula-fed infants^[119]^. Further studies of probiotic, prebiotic and synbiotic supplementation in the infant diet to ensure optimal microbial colonization of the infant gut are warranted.

### Genetics

Individuals with different genetic backgrounds harbour distinct microbes, as have been indicated by host and microbiome genome-wide association studies. These studies show similarities in the gut microbiome composition among phylogenetically-related individuals^[120]^. For instance, twin studies indicate that the microbiome of monozygotic twins is significantly closer than that of dizygotic twins^[121]^. Kurilshikov *et al.*^[122]^ reported an age-dependent association between the lactase (*LCT*) gene locus and the *Bifidobacterium *genus in the MiBioGen consortium. Indeed, the functional SNP in the *LCT *locus, rs4988235, has been shown to determine the abundance of the *Bifidobacterium *genus^[123]^. The DR4-DQ8 allele, the highest risk associated allele for type 1 diabetes, has also been hypothesised to serve as a gatekeeper for the presence or absence of gut bacteria in early life^[124]^. A study of dichorionic triplets suggested that host genetics initially play a major role in determining microbial community composition; however, by year one, environmental factors are the major determinant in healthy infants^[125]^, suggesting that nurture has the potential to overcome the genetic nature of an individual. Collectively, the findings of these studies demonstrated the influence of host genetics in the formation and development of the human microbiota. Further studies are needed to fully elucidate how genetic differences may shape the mother-to-infant microbiota transmission and the formation of the gut microbiome in the initial stages of life.

### Environment

Environmental factors such as place of birth and geographical location have also been reported to influence initial patterns of infant gut microbiota colonization. For instance, while the hospital setting contributes an antiseptic environment for labor and delivery, it also presents a possible exposure route of antibiotic-resistant bacteria, especially in the case of CS birth^[82]^. Lower beta diversity of *Bacteroides*, *Bifidobacterium*, *Streptococcus* and *Lactobacillus* and higher *Clostridium* and *Enterobacteriaceae* in hospital-born infants than babies delivered at home has been demonstrated^[126]^. Similarly, a study indicated that the microbiota of hospital-delivered infants was enriched with gram-positive anaerobic cocci, including the *Peptoniphilus* and *Finegoldia* genera, while those of home-delivered infants exhibited higher relative abundances of species from the *Enterococcus* and *Bifidobacterium* genera^[127]^. Geographical location and ethnicity are also proposed to impact the establishment of the infant gut microbiome; however, these microbiota differences seem to be associated with dietary and lifestyle patterns in a specific area or region^[128]^. For instance, children living in a rural village in Africa harbor a distinct microbiota to children residing in an urban region in Italy^[129]^. African infants show an increased relative abundance of bacilli, while North American infants have more Bacteroidetes and less Actinobacteria than infants in other continents^[79]^. Numerous additional studies have explored the geographical effect, as linked to ethnicity and/or diet, on microbial diversity and composition^[130]^.

## CONCLUSION

Evidence to date indicates that perinatal factors have a significant effect on the formation of the gut microbiota in the initial stages of life. Our understanding of exactly how such factors affect microbial colonization and, in turn, influence infant health is mostly limited to correlation rather than causation studies due to a previously underpowered toolkit. Furthermore, direct comparisons of studies of infant gut microbiota and perinatal factors have been limited due to the cultivation techniques used and the use of 16S rRNA amplicon sequencing, which have informed most of our understanding of the human microbiome to date. Yet, these approaches are hampered by the limited number of cultivable species^[131]^, primer design^[132]^, sample processing^[133]^, small sample sizes^[113]^ and the low taxonomic resolution provided by 16S rRNA gene profiling^[134]^. Most importantly, as 16S rRNA amplicon sequencing is only reliable to genus level, it does not provide information about the functional capacity of the microbiome. Fortunately, the advent of metagenomics sequencing (i.e., whole-genome shotgun sequencing) has illuminated much of the “dark matter” of our microbiome^[135]^ and will hopefully circumvent many of the limitations of previous studies. These methods allow characterization of microbial activity, helping us to elucidate not only which microbes are present, but also what metabolic functionalities these microbes possess. Such knowledge could uncover associations between the presence of particular microbes and particular neonatal diseases.

A deeper understanding of how perinatal factors may stimulate or hinder the inheritance and/or choice of microbes by infants in early life constitutes a viable strategy in the investigation of next-generation probiotics for therapeutic interventions that maintain/improve the health of infants. Large scale longitudinal human studies, with greater, equally balanced cohorts given the same strains and prescription of either prebiotics, probiotics or synbiotics at comparable times and by related delivery routes are needed to target the vertical transmission of maternal microbes to infants with regards to specific bacteria at higher taxonomic resolution (e.g., strains) and the gross changes of the infant’s microbiota.

Studies investigating how multiple perinatal factors may interact with each other in the formation of the infant gut microbiome are also warranted. For instance, breastfeeding has been shown to beneficially alter the patterns of aberrant colonisation in CS born infants^[136]^. Finally, detailed transcriptomics, metabolomics, lipidomics and proteomic investigations in combination with DNA and RNA sequencing will enable us to fully uncover the effects of perinatal factors on initial microbiome establishment and long-term functioning.

## DECLARATIONS

### Copyright

© The Author(s) 2022.
